# Stanniocalcin-2 promotes cell EMT and glycolysis via activating ITGB2/FAK/SOX6 signaling pathway in nasopharyngeal carcinoma

**DOI:** 10.1007/s10565-021-09600-5

**Published:** 2021-04-02

**Authors:** Jingquan Li, Zihao Zhang, Xu Feng, Zhuqing Shen, Ji Sun, Xiuwen Zhang, Fengjiao Bu, Midie Xu, Cong Tan, Ziliang Wang

**Affiliations:** 1 Center for Single-Cell Omics, School of Public Health, Shanghai Jiao Tong University School of Medicine, Shanghai, 200025 China; 2 Clinical Research Unit of Shanghai municipal Hospital of Traditional Chinese Medicine, Shanghai University of Traditional Chinese Medicine, 274 Zhijiang Middle Road, Shanghai, 200071 China; 3 School of Pharmacy, Anhui Medical University, 81 Meishan Road, Hefei, 230032 Anhui China; 4 Department of Pathology and Tissue Bank, Fudan University Shanghai Cancer Center, 270 Dong’an Road, Shanghai, 200032 China; 5 Department of Pharmacy, Eye & ENT Hospital, Fudan University, Shanghai, 200031 China

**Keywords:** Nasopharyngeal carcinoma, EMT, Glycolysis, STC2

## Abstract

Stanniocalcin-2 (STC2) has been proved to regulate a variety of signaling pathways including cell growth, metastasis, and therapeutic resistance. However, the role of STC2 in the regulation of nasopharyngeal carcinoma (NPC) remains poorly understood. In this study, we investigated the regulatory function of STC2 on epithelial-mesenchymal transition (EMT) and glycolysis traits in NPC and revealed the underlying molecular mechanisms. We found that STC2 was highly expressed in primary nasopharyngeal carcinoma tissues and lymph node metastatic tissues. Silencing of STC2 inhibited cell proliferation, invasion, and glycolysis. Further analyses for the clinical samples demonstrated that STC2 expression was associated with the poor clinical progression. Moreover, we demonstrated the interaction of ITGB2 with STC2 and its involvement in STC2-mediated ITGB2/FAK/SOX6 axis. Collectively, our results provide new insights into understanding the regulatory mechanism of STC2 and suggest that the STC2/ITGB2/FAK/SOX6 signaling axis may be a potential therapeutic target for NPC.

**Supplementary Information:**

The online version contains supplementary material available at 10.1007/s10565-021-09600-5.

## Introduction

Nasopharyngeal carcinoma (NPC) is a malignant epithelial tumor associated with Epstein–Barr virus (EBV) infection (Strazzulla et al. 2015; Raab-Traub 2002). The incidence of NPC varies with obvious geographical distribution characteristics; 70% of the new cases occur in East Asia and Southeast Asia, especially Southern China (Lo et al. 2004). Although the overall survival of NPC patients has been significantly improved with the advancement of radiotherapy technology and chemotherapy, a large proportion of NPC patients still developed recurrence or distant metastasis due to the radioresistance or late recognition (Lee et al. 2005). The failure of these treatments greatly threatens the patient’s long-term survival and the quality of life. Studies have shown that the abnormal expression of genes or proteins can affect the growth, metastasis, or therapeutic effect of NPC (Zhao et al. 2018). Therefore, exploring the mechanism of NPC development and metastasis and identification of effective anti-NPC factors have been emerging as a great significance for the treatment of NPC.

Epithelial-mesenchymal transition (EMT) is a process that epithelial cells lose adhesion ability, polarity, and remodeling of the cytoskeleton (Thiery 2002). Along with the weakening of epithelial cell adhesion ability and the loss of cytoskeletal components, cells undergoing epithelial transformation acquire mesenchymal component expression and migration capability. This is usually a hallmark of cancer. In addition, glycolysis is another hallmark of cancer. Due to the Warburg effect, cancer cells rely on glycolysis for energy supply in the presence of oxygen and produce large amount of lactic acid and ATP (Hanahan and Weinberg 2011; Koppenol et al. 2011). Recent studies have shown that EMT and glycolysis are closely related to the growth and metastasis of NPC as well; inhibition of NPC EMT and glycolysis could significantly suppress the growth and metastasis of NPC (Cai et al. 2018; Lyu et al. 2018; Yu et al. 2019; Horikawa et al. 2007, 2011; Kong et al. 2010).

Stanniocalcin-2 (STC2) is a secretory homodimer glycoprotein, which can be expressed in a variety of tissues and has autocrine, paracrine, and endocrine functions. Its C-terminal contains histidine residues that interact with metal ions. STC2 involves in regulating cell metabolism, calcium/phosphate transport, and homeostasis (Ishibashi et al. 1998). In addition, STC2 plays an important role in the development of cancers (Esseghir et al. 2007; Ieta et al. 2009; Meyer et al. 2009; Tamura et al. 2009; Law and Wong 2010; Kita et al. 2011; Wu et al. 2017; He et al. 2019; Ke et al. 2019), including hepatocellular carcinoma, head and neck squamous cell carcinoma, lung cancer, laryngeal squamous cell carcinoma, breast cancer, and ovarian cancer. STC2 is a HIF-1α target gene and Law et al. found that STC2 promotes cancer invasion and metastasis in hypoxic cells (Law and Wong 2010). Kita et al. revealed that STC2 is associated with lymph node metastasis in esophageal cancer patients (Kita et al. 2011) while high STC2 expression showed significantly worse overall survival rates in colorectal cancer (Ieta et al. 2009).

In this study, we tried to reveal the potential function and mechanisms of STC2 in NPC. We not only found its high expression in primary nasopharyngeal carcinoma tissues and lymph-node metastatic tissues, but also found that STC2-mediated regulation of EMT and glycolysis traits was partially realized through the modulation of ITGB2/FAK/SOX6 signaling. We demonstrated the direct interaction between STC2 and ITGB2, and then FAK as the downstream of ITGB2 was activated, which led to increased SOX6 expression. The elevation of SOX6 expression not only regulated the EMT-related gene expression, but also enhanced glycolysis-related gene expression at the transcriptional level. Combining with the data from the clinical samples demonstrated that STC2 expression was associated with the poor clinical progression. Our findings may provide new insights into understanding and exploring new therapeutic targets for NPC treatment.

## Methods and materials

### Tissue samples

Formalin-fixed paraffin-embedded (FFPE) tissues of 68 NPC patients who underwent surgery in Shanghai Cancer Center of Fudan University from June 2010 to January 2019 were studied by immunohistochemistry. None of them received drug treatment before operation. The follow-up period was from the date of diagnosis to the date of the most recent clinical investigation, disease progression, or death. This study was ethically approved by the FUSCC Ethics Committee, and all patients provided a written informed consent form. Histological examination was performed by an experienced pathologist to determine the location of all lesions. Each case has two different cores to rule out tumor heterogeneity. Two suitable cancer foci and one normal epithelial paracancerous focus were randomly selected from 68 patients and the recipient paraffin blocks were implanted.

### Cell culture and transfection

Human nasopharyngeal carcinoma cell line 6-10B and CNE1 were purchased from ATCC cell bank. These cells were cultured in DMEM medium (Hyclone, SH30243.01) containing 10% fetal bovine serum (Gibco, FBS) and 1% antibiotics. All cells were grown in a 37 °C incubator with 5% CO_2_. Stable silencing of STC2 expression was accomplished using lentivirus of STC2 shRNA. Polybrene (10 μg/ml) was added to the cells for 12 h, then the medium was changed, and the cells expressing puromycin resistance were screened by puromycin (Shanghai MaoKang Biotechnology, MS0011-25MG). The cells expressing puromycin resistance could survive in the culture medium. The ITGB2 plasmid was incubated with Lipofectamine 2000 (Thermo Fisher Scientific, 11668019) for 15 min and then added to 80% of the confluent cells, and the medium was changed after 6 h.

### qRT-PCR

Total RNA was extracted using TRIzol and was transcribed into cDNA using a PrimeScript RT-PCR kit (Takara, RR014A). The primer sequences are listed in Supplementary Table 1.

### Western blot

Ripa lysis buffer (Solarbio, R0020) was used to obtain cell lysis. BCA protein assay kit (Thermo Fisher, Rockford, IL, USA) was used for protein quantification. Twenty micrograms of protein extracts was electrophoretically on 10% SDS-PAGE gels. Next, the proteins were transferred to polyvinylidene fluoride membranes (Millipore, IPVH00010). After blocking with 5% non-fat milk for 2 h, the membranes were incubated with primary antibodies (Supplementary Table 2) at 4 °C overnight and subsequently incubated with a secondary HRP-conjugated antibody for 1 h at room temperature. The protein signals were detected using enhanced chemiluminescent HRP substrate (Merck Millipore).

### RNA-seq data analysis

Total RNA (1 μg) was isolated from CNE1 cells and treated with VAHTS mRNA Capture Beads (Vazyme, Nanjing, China) to enrich polyA+ RNA before constructing the RNA libraries. RNA library preparation was performed by using VAHTS mRNA-seq v2 Library Prep Kit from Illumina (Vazyme, Nanjing, China). Paired-end sequencing was performed with Illumina HiSeq 3000 at RiboBio Co., Ltd. (Guangzhou, China). For computational analysis of RNA-seq data, sequencing reads were aligned using the spliced read aligner HISAT2, which was supplied with the Ensemble human genome assembly (Genome Reference Consortium GRCh38) as the reference genome. Gene expression levels were calculated by the fragments per kilobase of transcript per million mapped reads (FPKM). Gene Set Enrichment Analysis (GSEA) was used for gene functional annotation.

### PET/CT

The whole-body 18F-FDG positron emission tomography/computed tomography imaging (PET/CT) was performed as previously described. Animals fasted for at least 12 h to ensure low levels of serum glucose. Scanning started 1 h after intravenous injection of the tracer (7.4 MBq/kg). The images were acquired on a Siemens biograph 16HR PET/CT scanner with a transaxial intrinsic spatial resolution of 4.1 mm. Quantification of metabolic activity was acquired using the standard uptake value (SUV) normalized to body weight, and the SUVmax for each lesion was calculated.

### Immunoprecipitation

Immunoprecipitation (IP) was performed using Pierce Crosslink Immunoprecipitation Kit (Thermo, 26147) following the manufacturer’s protocol. Cells were lysed in RIPA lysis buffer (25 mM Tris-HCl, pH 7.4, 0.15 M NaCl, 0.001 M EDTA, 1% NP-40, 5% glycerol) with the presence of a protease inhibitor cocktail mixture (Sigma-Aldrich). Cells lysates were pre-cleared by the control agarose resin and then immunoprecipitated using anti-STC2 antibody (Abcam, ab255610) overnight at 4 °C. Antigen was eluted and subjected to SDS page electrophoresis.

### Cell viability assay

5×10^3^ cells were seeded in the 96-well plate. On the second day, the cells were treated with different concentrations of cisplatin (0, 2, 4, 8, 16, 32, 64, 128μg/ml). CCK8 (TargetMol, C0005) was added 48 h later, and after incubation for 2 h, the absorbance at 450 nm was determined by enzyme labeling instrument. Each group contains three composite holes.

### Cell proliferation assay

2×10^3^ cells were seeded in the 96-well plate. After the cells were adhered, CCK8 were added in 24 h, 48 h, 72 h, and 96 h to detect the cells viability. The absorbance at 450 nm was determined by enzyme labeling instrument. Each group contains three composite holes.

### Colony formation assay

The cells were seeded in a 6-well plate with 5×10^2^ in each well and placed in a 37 °C incubator containing 5% CO_2_ for 12 days. After the community formation was observed, the abandoned culture medium was washed with PBS (Hyclone, SH30256.01), fixed with 4% paraformaldehyde (Beyotime, P0099), and stained with crystal violet (Beyotime, C0121).

### Invasion and migration assay

In the migration experiment, cells (5×10^4^) were added to the upper cavity of transwell (Corning, 3422) chamber with FBS-free DMEM. The lower chambers were filled with 500 μl DMEM containing 20% FBS. After the chamber was taken out, it was fixed with 4% paraformaldehyde, stained with crystal violet, erased the cells retained in the upper part of the membrane, and counted under the microscope. The invasion experiment is similar to the migration experiment, except that the upper cavity of the transwell chamber was covered by Matrigel (BD Biosciences).

### Wound healing assay

Spread the cells in a 6-well plate until the cells are fully fused, draw a straight line with an aseptic suction head, and wash the cell fragments with PBS. After 0 h, 12 h, and 24 h, respectively, the healing ability of the scratched cells was observed under microscope.

### Immunofluorescence and confocal microscopy

Spread the cells in a 6-well plate with glass slides until the cell confluence reaches 70%, fix them with 4% paraformaldehyde for 30 min, seal them with immunofluorescence sealing solution (Beyotime, P0102) at room temperature for 2 h, then incubate them with primary antibodies (Supplemental Table 2) overnight at 4 °C. On the second day, incubate with second antibody (1 : 1000) for 1 h, then stain with DAPI for 5 min, wash off with PBS, and photograph under a microscope.

### Immunohistochemistry

Clinical tissue samples were obtained from Fudan University Shanghai Cancer Center (FUSCC). The expression of STC2, ITGB2, and SOX6 in human tissue and the expression of STC2, ITGB2, and SOX6 in mouse tissue were detected by immunohistochemistry (IHC). The stained sections were evaluated and scored independently by two pathologists. Staining was scored based on maximal staining intensity (0, negative; 1, weak; 2, moderate; 3, strong), and the percentage of positively stained cells (0, no staining; 1, <10% of cells; 2, 11–50% of cells; 3, 51–80% of cells; and 4, >81% of cells stained).

### Animal model

All animal experiments were approved by the Institutional Animal Care and Use Committee of Fudan University and performed according to institutional guidelines. Female BALB/c nude mice (Shanghai SLAC Experimental Animal Co., Ltd., 4–6 weeks) were randomly divided into three groups and fed in SPF animal room. Each group was subcutaneously injected with CNE1-STC2-control, CNE1-STC2-sh1, and CNE1-STC2-sh2 cells (5×10^6^ cells each mouse, suspension was 0.1 mL PBS). The size of tumor was measured with vernier caliper and positron emission tomography (PET/CT) was performed. The standard uptake value (SUV) was used to evaluate the glucose uptake of the tumor. The mice were raised for 30 days, and the tumor tissue was removed and fixed in 4% paraformaldehyde.

### Glycolysis analysis

The glycolysis process was tested by the Lactate Colorimetric Assay Kit (BioVision), Glucose Uptake Colorimetric Assay Kit (BioVision), Amplite Colorimetric NADPH Assay Kit (AAT Bioquest Inc.), and ATP Assay Kit (SIGMA ALOR-ICH) following the manufacturer’s protocols.

### Xenograft tumor zebrafish model

Wild-type AB zebrafish (female and male) were purchased from China zebrafish Resource Center. Under the standard circulating water system, the light/dark cycle of 14 h/10 h was kept at 28 °C, and the PH value was kept between 7.0 and 8.0. The male fish and the female fish (2:3) were naturally mated in a water tank to obtain embryos. CNE1 cells were labeled with cell tracer DIO. The cells were washed with PBS, then digested and centrifuged. DIO-labeled CNE1 cells were transferred into the yolk sac of each zebrafish embryo using a microsyringe. A total of 20 to 30 fluorescent cells are injected into the yolk of zebrafish. The embryos were incubated at 36 °C for 3 days and analyzed by fluorescence microscope (OLYMPUS).

### Statistical analyses

The data are presented as mean ± SD in the figures. A Student *t* test was used to analyze the data. Kaplan-Meier analysis was used to analyze overall survival and progression-free survival. *P* ≤ 0.05 were considered statistically significant.

## Results

### STC2 is involved in tumorigenesis and metastasis

**Fig. 1:**
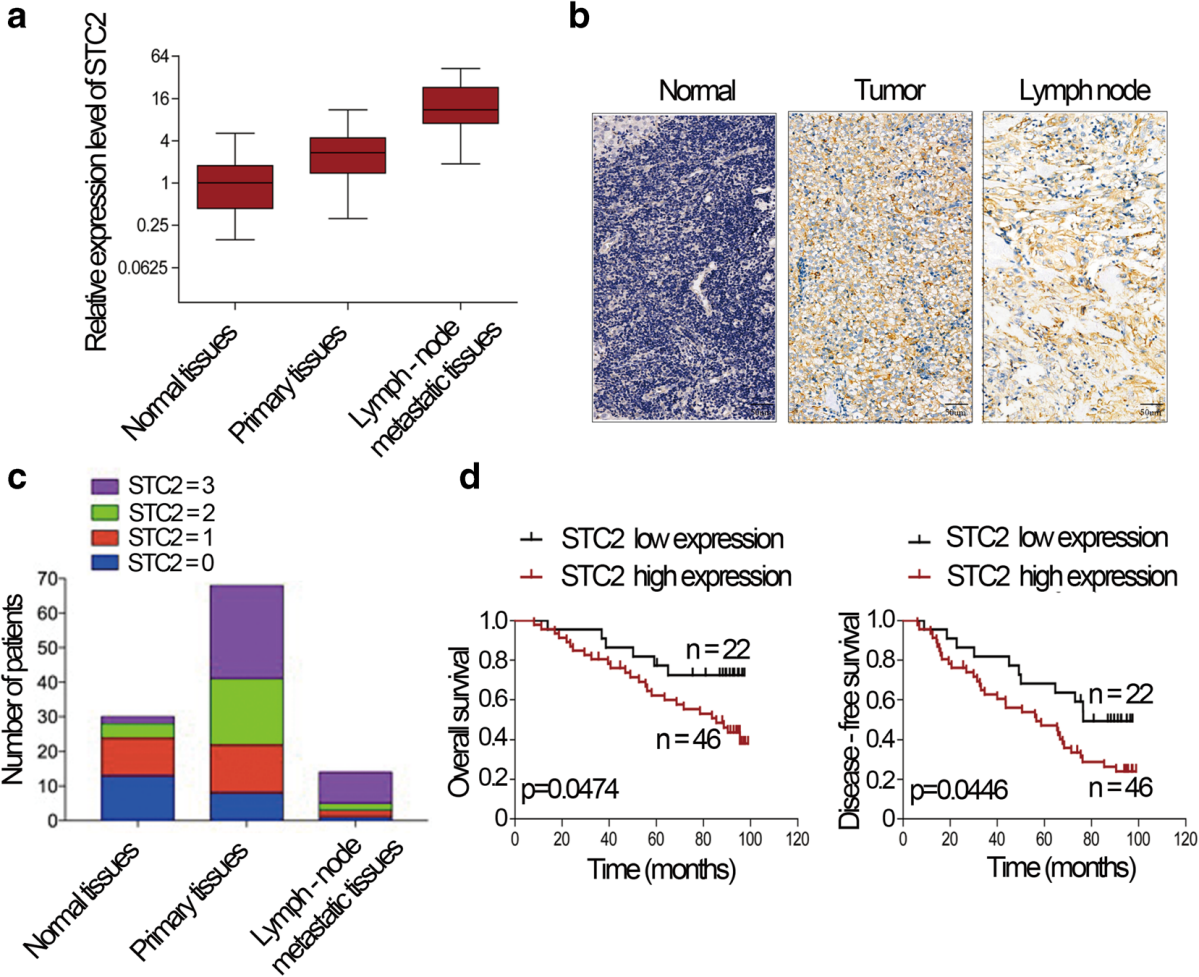
STC2 is involved in tumorigenesis and metastasis. **a** Expression of STC2 in normal tissues, primary tissues, and lymph node metastatic tissues of human nasopharyngeal carcinoma. **b** IHC analysis and positive distribution of STC2 in normal tissues, primary tissues, and lymph node metastatic nasopharyngeal carcinoma tissues. **c** IHC scores of STC2 in different types of tissues. **d** Kaplan-Meier survival curve showed that the OS and DFS of STC2 high expression group exhibit a poor progression than those of low expression group

To assess the role of STC2 in human nasopharyngeal carcinoma, we first determined the expression levels of STC2 in normal tissues, primary carcinoma tissues, and lymph-node metastatic tissues of human nasopharyngeal carcinoma. The results showed that the STC2 was upregulated in primary nasopharyngeal carcinoma tissues than normal tissues. Furthermore, STC2 was also significantly upregulated in lymph node metastasis than nasopharyngeal carcinoma tissues (Fig. 1a). Immunohistochemistry results showed that STC2 staining of the lymph node metastatic and primary cancer tissues was obviously stronger than that of normal tissues (Fig. 1b). The scores of IHC was shown in Fig. 1c. Then, we analyzed the association of nasopharyngeal carcinoma patient survival with STC2 expression. We found that poor overall survival (OS) and disease-free survival (DFS) were significantly associated with STC2 expression (Fig. 1d). These results indicate that STC2 is upregulated in nasopharyngeal carcinoma and involved in tumorigenesis and metastasis.

### STC2 participates in EMT and glycolysis pathway

**Fig. 2:**
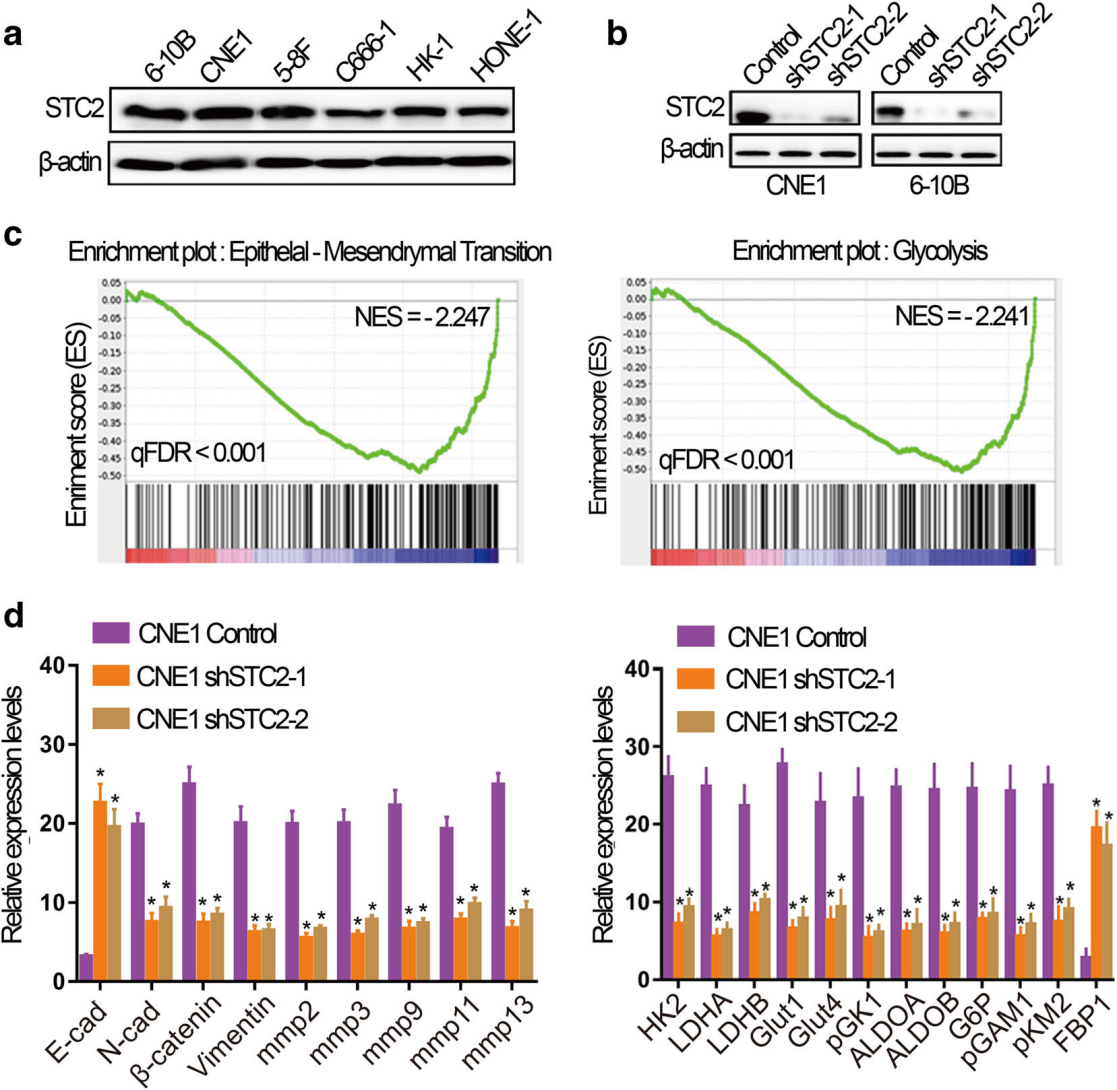
STC2 participates in EMT and glycolysis pathway. **a** The expression level of STC2 detected by western blotting in nasopharyngeal carcinoma cell lines, including 6-10B, CNE1, 5-8F, C666-1, HK-1, and HONE-1 cells. **b** The validation of STC2 knockdown efficiency by western blotting. **c** Analysis of cell function regulated by RNA-seq. **d** qRT-PCR analysis of indicated genes in STC2 knockdown and its control cells. Data are shown as mean ± SD. Significance was calculated using Student’s *t* test. *p < 0.01

To explore the function of STC2 in NPC, we first analyzed the expression level of STC2 in different NPC cell lines by Western blot and found that STC2 was comparatively highly expressed in 6-10B and CNE1 (Fig. 2a). So we chose 6-10B and CNE1 to knock down STC2. The knockdown efficiency of STC2 in 6-10B and CNE1 is validated in Fig. 2b. To explore the effect of STC2 on the pathogenesis of nasopharyngeal cancer, we performed RNA-seq on STC2 knockdown cell line CNE1 and its control cells. The results showed that the genes that were downregulated after STC2 knockdown were enriched in EMT and glycolysis pathway (Fig. 2c). Subsequently, we performed qRT-PCR to validate the results. As shown in Fig. 2d, STC2 knockdown decreased EMT-related genes, such as N-cadherin, β-catenin, and MMPs while it increased E-cadherin expression. Meanwhile, STC2 knockdown also inhibited the expression of glycolysis-related genes, such as LDHA, LDHB, GLUT1, GLUT4, PGK1, ALDOA, ALDOB, G6P, PGAM1, and PKM2 while it enhanced FBP1 expression. Taken together, STC2 can participate in EMT and glycolysis in nasopharyngeal cancer by regulating their related genes.

### The effect of STC2 on the growth and metastasis

**Fig. 3:**
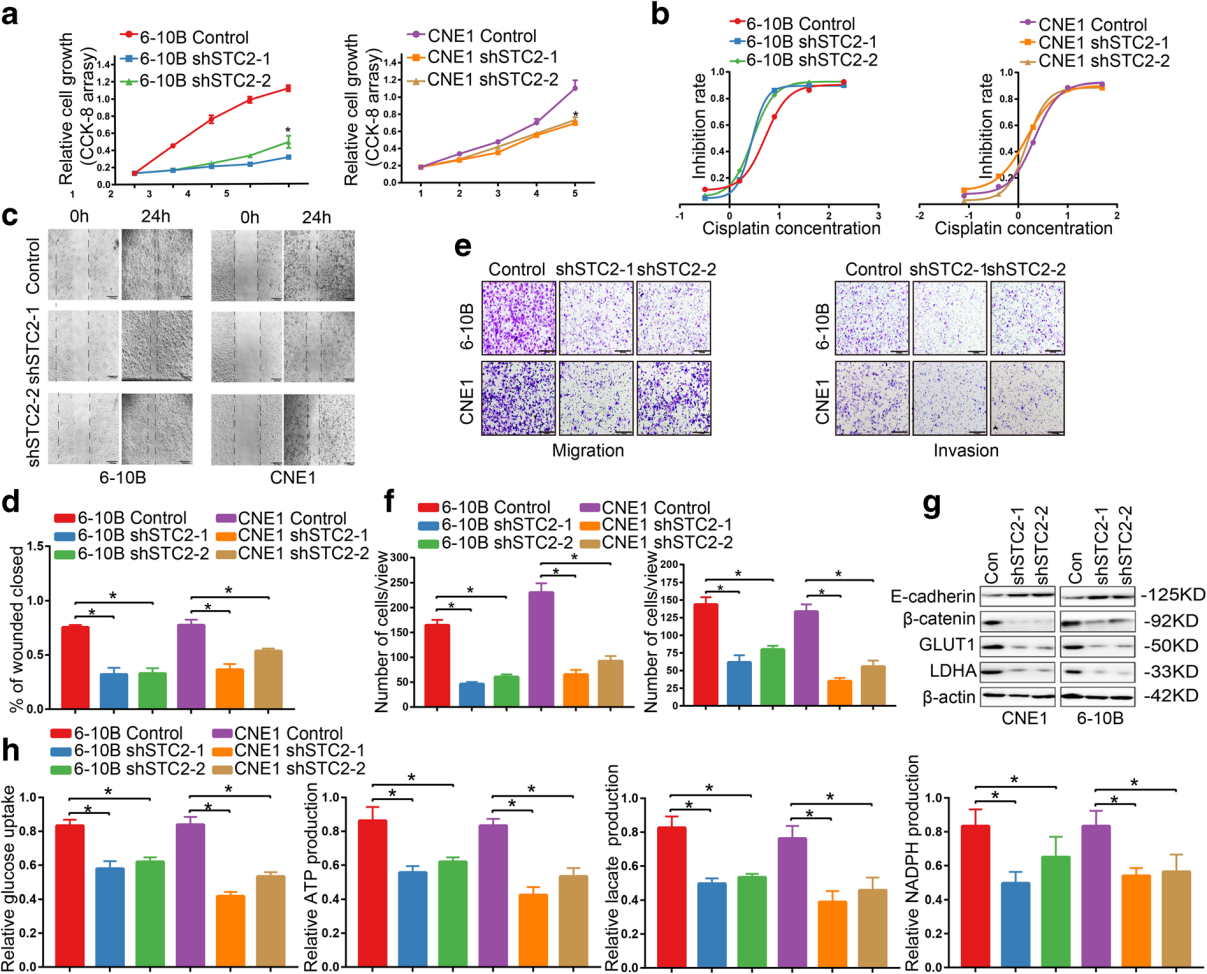
The effect of STC2 on the growth and metastasis. **a** CCK-8 assays showed that knockdown of STC2 suppressed cells proliferation in 6-10B and CNE1 cells. *p < 0.01. **b** Drug sensitivity test showed that STC2 knockdown increased drug sensitivity in 6-10B and CNE1 cells. **c**, **d** Evaluation of cell migration ability through wound healing analysis. *p < 0.01. **e**, **f** Evaluation of cell migration and invasion ability through transwell analysis. *p < 0.01. **g** Effect of STC2 knockdown on the expression level of key EMT factors and glycolytic enzymes by western blotting. **h** The glucose uptake/ATP/lactate/NADPH production were significantly repressed in STC2 knockdown cells compared to its control cell lines *p < 0.01

To explore the function of STC2 in nasopharyngeal cancer, we detected the cell viability by CCK-8 and found that STC2 knockdown led to decreased proliferative capacity in 6-10B and CNE1 cells (Fig. 3a). Furthermore, STC2 knockdown 6-10B and CNE1 cells were more sensitive to cisplatin treatment (Fig. 3b), displaying a significantly reduced IC50 of cisplatin, compared to the its control cells (Fig. 3b), suggesting the enhanced anti-tumor effect for chemotherapeutic treatment upon STC2 silencing (6-10B control IC50=5.2μM, 6-10B shSTC2-1 IC50=2.74μM, 6-10B shSTC2-2 IC50=2.9μM, CNE1 control IC50=2.14μM, CNE1 shSTC2-1 IC50=1.46μM, CNE1 shSTC2-2 IC50=1.48μM). To investigate whether STC2 expression could affect the migration ability, we performed scratch and transwell assay. As shown in Fig. 3c–f, the migration speed of STC2 knockdown cells was slowed down after 24 h culture and the invasion ability was also reduced. Furthermore, colony formation assay indicated that STC2 knockdown caused the morphology changes of 6-10B and CNE1 cells with a spindle-shaped boundary turning to a round shape (Supplementary Figure 1A). The expression level of key EMT factors and glycolytic enzymes were also detected by western blot on STC2 knockdown cells. The results showed that STC2 knockdown increased the expression of E-cadherin while it reduced the expression of β-catenin, GLUT1, and LDHA (Fig. 3g). To further assess the role of STC2 in glycolysis, we performed glucose uptake, ATP production, lactate production, and NADPH production assays in 6-10B and CNE1 cells. We found that the glucose uptake and ATP/lactate/NADPH production were significantly repressed in STC2 knockdown cells compared to its control cell lines (Fig. 3h).

### Effect of knockdown STC2 on the downstream molecules

**Fig. 4:**
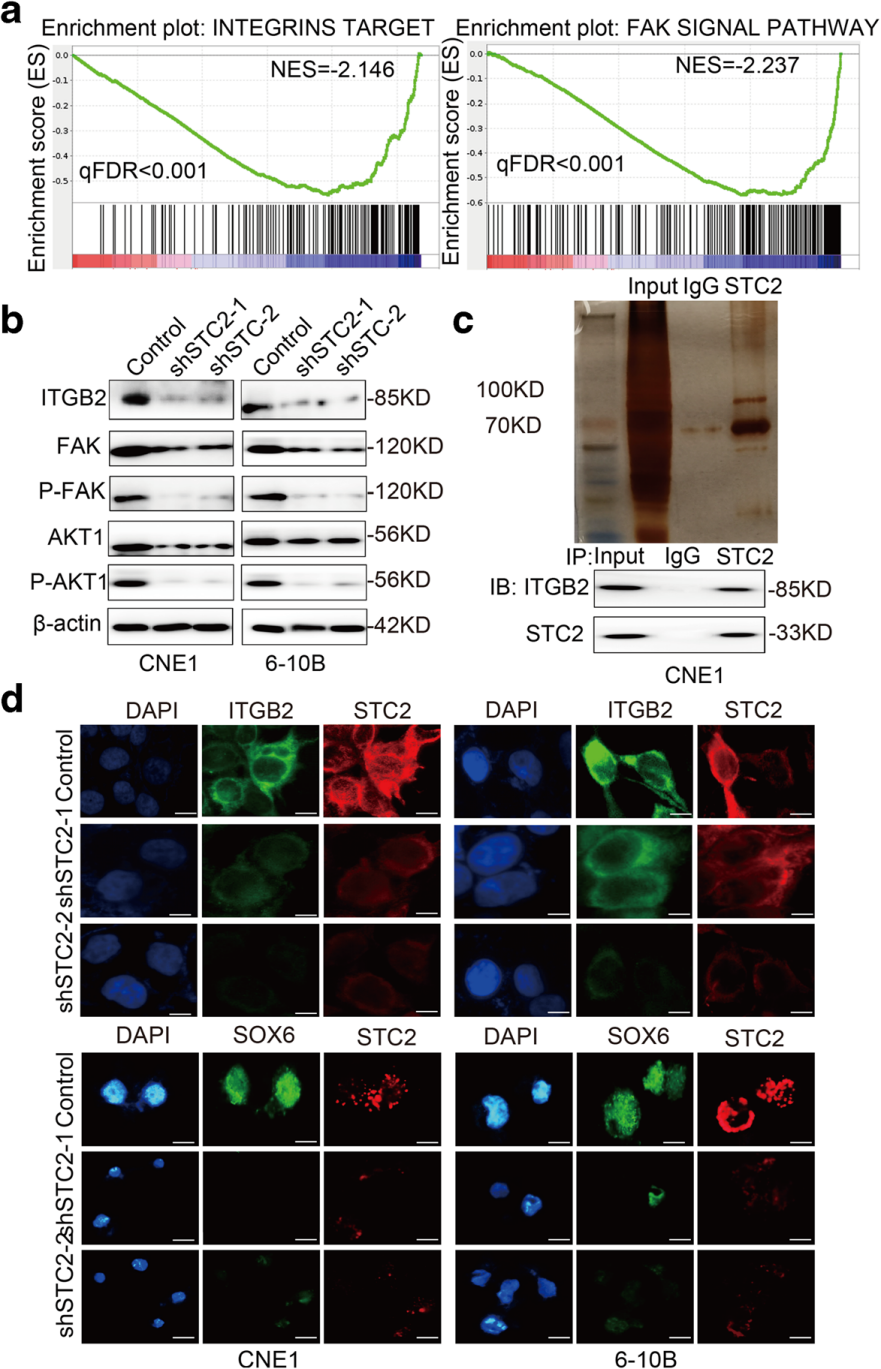
Effect of knockdown STC2 on the downstream molecules. **a** GSEA analysis of STC2 knockdown RNA-seq data. The signature was defined by genes with significant expression changes. **b** Western blotting was used to detect the effect of STC2 knockdown on the expression of ITGB2, FAK, P-FAK, AKT1, and P-AKT1. **c** Co-immunoprecipitation and silver staining were performed to detect the potential proteins interact with STC2. **d** Representative immunofluorescent images of STC2, ITGB2, and SOX6 in STC2-silencing cell lines

To further explore the mechanism of STC2 in nasopharyngeal cancer, we analyzed GSEA results upon STC2 knockdown RNA-seq data and identified integrins and FAK signal pathway as potential downstream targets (Fig. 4a). The autocrine or paracrine abilities of STC2 and previous reports have shown that the integrin-FAK signal could activate many signal pathways through phosphorylation and protein interaction to promote tumorigenesis^25, 26, 27, 28^. Thus, we focused on this pathway and detected the protein expression level related to those pathways. The results showed that knockdown of STC2 led to the reduced expression of ITGB2, FAK, p-FAK, AKT1, and p-AKT1 (Fig. 4b). Subsequently, we used co-immunoprecipitation to pull down the potential proteins which interact with STC2 protein. To our surprise, mass spectrometry analysis identified ITGB2 specifically interacting with STC2 and western blot was used to validate this result (Fig. 4c). Immunofluorescent imaging also showed that STC2-silencing suppressed the expression of ITGB2 (Fig. 4d). Then, we performed rescue experiments of ITGB2 in STC2 knockdown cells and found that ITGB2 overexpression reversed STC2-silencing mediated the inhibition of migration and invasion (Supplementary Figure 1B and 1C).

### SOX6 is the downstream target of STC2

**Fig. 5:**
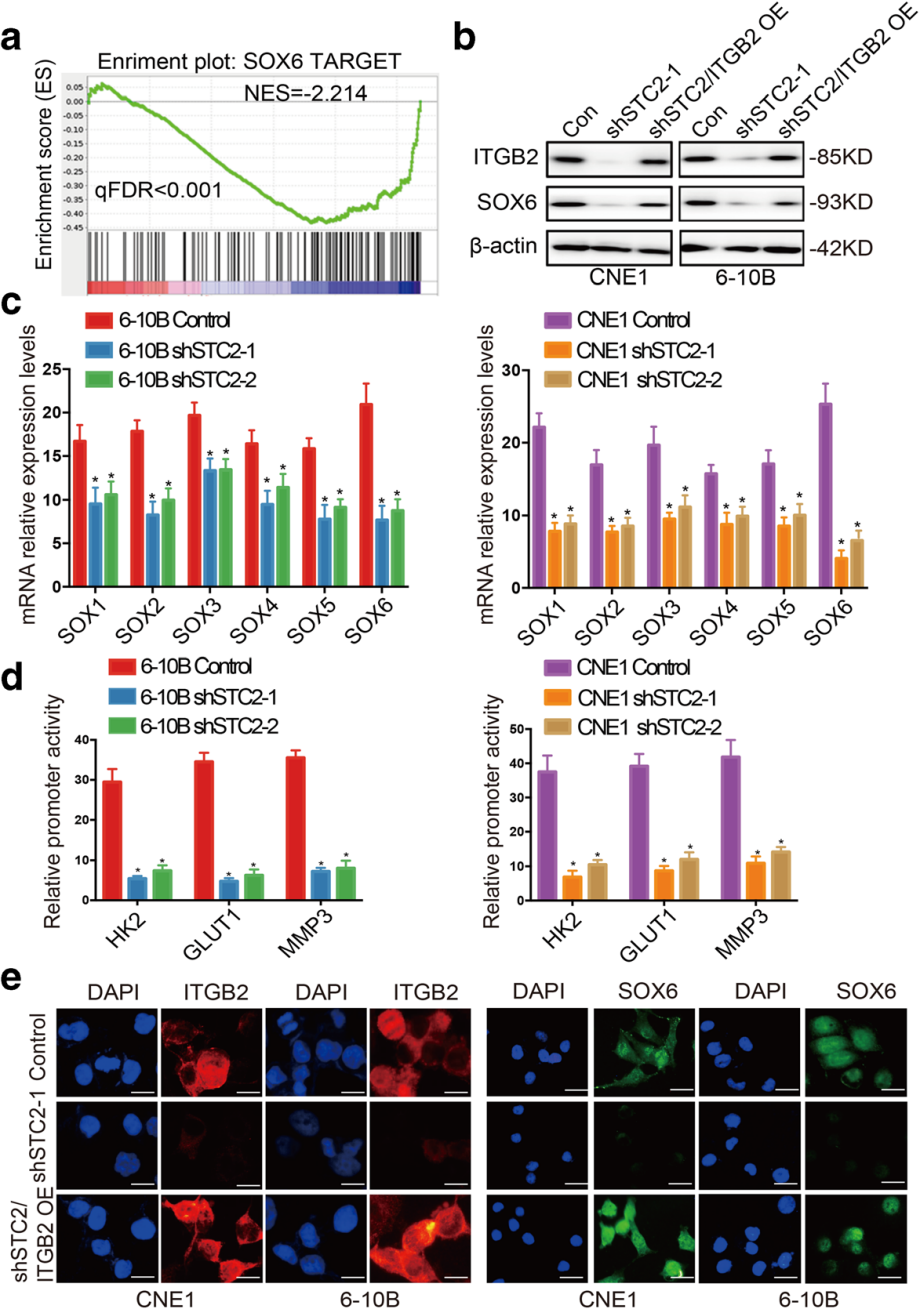
SOX6 is the downstream target of STC2. **a** GSEA analysis of STC2 knockdown RNA-seq data. The signature was defined by genes with significant expression changes. **b** The effects of STC2 knockdown and ITGB2 rescue were detected by Western blotting. **c** qRT-PCR analysis of SOX-related genes in STC2 silencing and its control cell lines. Data are shown as mean ± SD. Significance was calculated using Student’s *t* test. *p < 0.01. **d** Compared with control group, STC2 knockdown affected the activity of HK2/GLUT1/MMP3 promoter activity in 6-10B and CNE1 cells. **e** Immunofluorescence staining showed the inhibition of SOX6 expression caused by STC2 could be rescued by ITGB2 overexpression

According to the GSEA results, STC2 silencing also suppressed SOX6 target signaling (Fig. 5a). Furthermore, the inhibition of SOX6 expression caused by STC2 could be rescued by ITGB2 overexpression (Fig. 5b, e). qRT-PCR also showed that SOX-related genes in STC2 knockdown cells were inhibited (Fig. 5c). Previous studies showed that the SOX family, including SOX6, has the ability to regulate glycolysis and EMT key factors’ promoter activity^29, 30, 31, 32^. So, we speculated whether STC2’s regulation of EMT and glycolysis is realized on SOX6. Therefore, we detected the influence of STC2 on SOX6-targeted genes’ promoter activity, including HK2/GLUT1/MMP3. As shown in Fig. 5d, compared with control group, STC2 knockdown affected the activity of HK2/GLUT1/MMP3 promoter activity in 6-10B and CNE1 cells.

### STC2 inhibits the proliferation of nasopharyngeal carcinoma cells in vivo

**Fig. 6:**
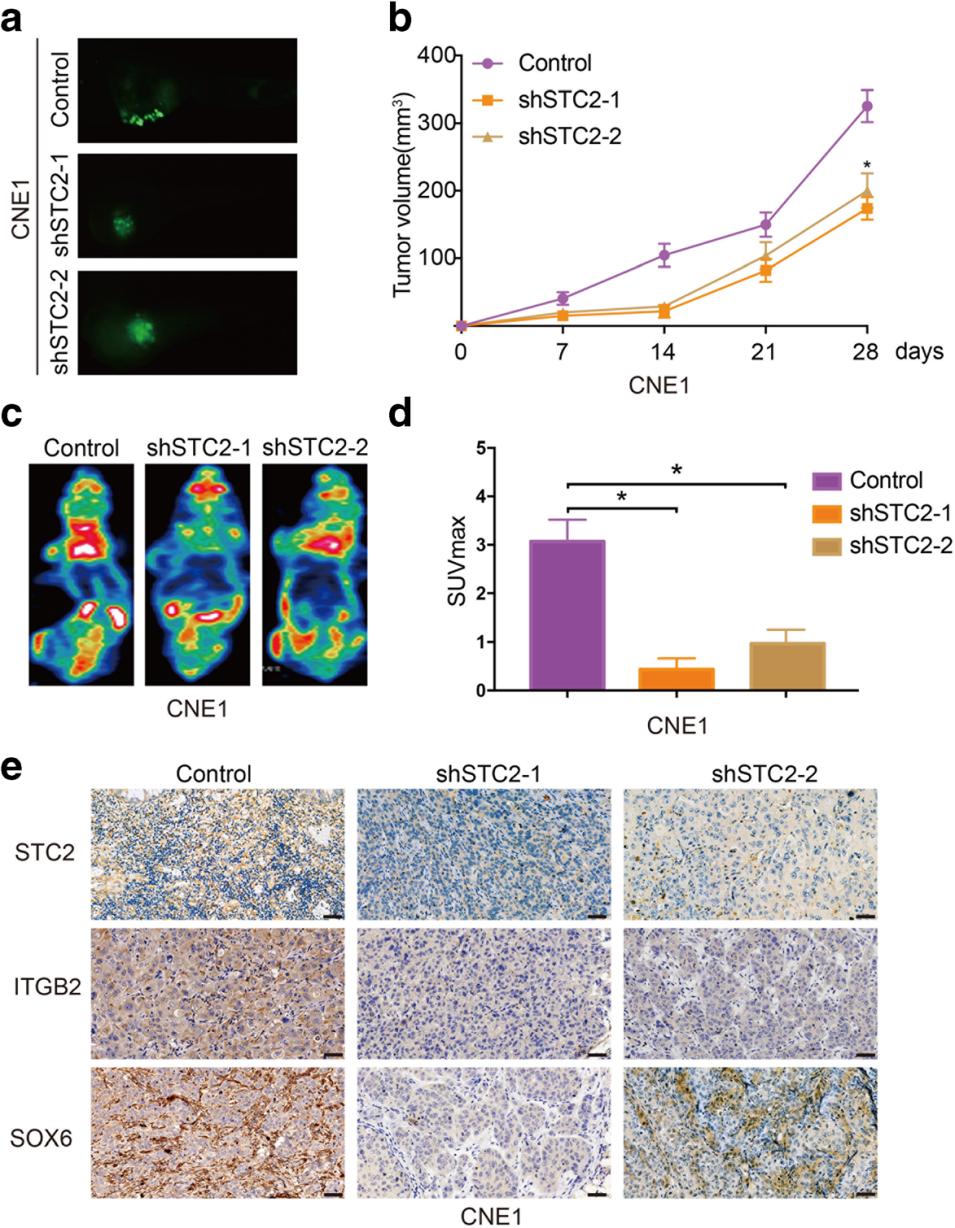
STC2 inhibits the proliferation of nasopharyngeal carcinoma cells in vivo. **a** Zebrafish embryo nasopharyngeal cancer model for determine the tumorigenesis of STC2. **b** Tumor diameters were measured and the tumor volume was calculated. **c** Representative image of PET-CT. **d** Average SUVmax values of nude mice bearing tumors. **e** Representative images of Immunohistochemistry staining of STC2, ITGB2, and SOX6

In order to further clarify whether STC2 is involved in tumor growth, the STC2 knockdown cell line CNE1 and its control cell line were used to establish the zebrafish embryo nasopharyngeal cancer model and nude mice model. We use the zebrafish model as a simple and effective system to test the proliferation ability of CNE1 cells. We monitored the activity of CNE1 cells and changes of SIV in zebrafish embryo nasopharyngeal cancer model by the use of epifluorescent microscopy and found that STC2 knockdown obviously suppressed the proliferate of cancer cells in zebrafish (Fig. 6a). Nude mice model results showed that knockdown of STC2 significantly inhibited tumor growth (Fig. 6b). The tumor weight of the STC2 knockdown group was smaller than that of the control cell group (Supplementary Figure. 2A). PET/CT results also revealed that STC2 knockdown significantly suppressed in vivo glucose uptake in CNE1 cells and caused a lower SUVmax value (Fig. 6c, d). Subsequent IHC analyses revealed that STC2 knockdown decreased the expression of ITGB2 and SOX6 in the xenografted tumor tissues (Fig. 6e).

### STC2 positively correlates with ITGB2/SOX6 and indicates a poor progression in clinical patients

**Fig. 7:**
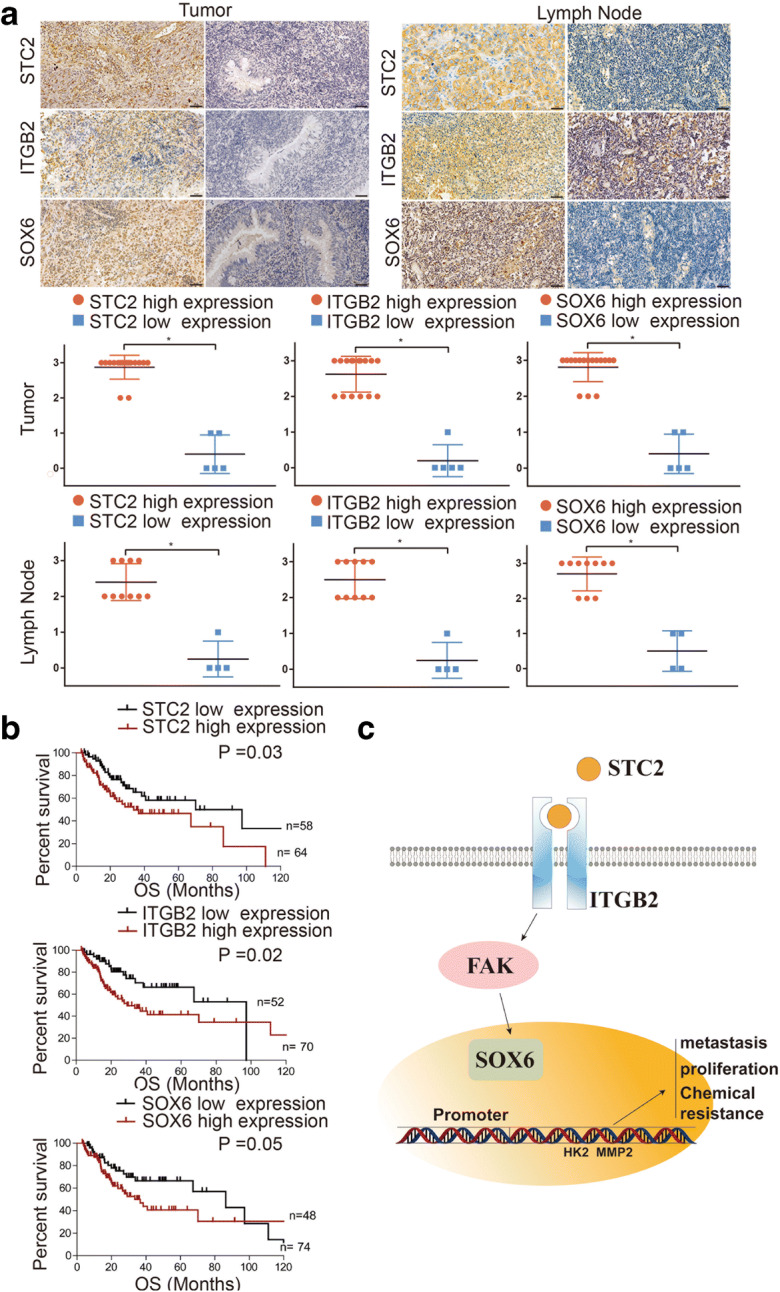
STC2 positively correlates with ITGB2/SOX6 and indicates a poor progression in clinical patients. **a** Representative images of IHC staining of STC2, ITGB2, and SOX6 in primary tissues and metastatic tissues. **b** The influence of STC2, ITGB2, and SOX6 on the overall survival in nasopharyngeal carcinoma. **c** Schematic model showing the role of the STC2/ITGB2/FAK/SOX6 signaling axis in the regulation of metastasis proliferation and chemical resistance

Next, we studied the clinical significance of STC2-ITGB2-SOX6 axis in patients with nasopharyngeal carcinoma. We divided the clinical samples into two groups, high and low expression of STC2, and found that the expression of ITGB2 and SOX6 was also high in STC2 high expression group both in primary tissues and in metastatic tissues (Fig. 7a). Then, we explored the prognostic value of STC2/ITGB2/SOX6 in nasopharyngeal carcinoma. Kaplan-Meier analysis demonstrated that higher STC2/ITGB2/SOX6 expression was associated with poor overall survival and progression-free survival (Fig. 7b and Supplementary Figure 2B). Based on the results from our study, we propose a schematic diagram of STC2 in the regulation of nasopharyngeal carcinoma. Taken together, our results demonstrated that STC2 interacted with ITGB2 to activate its downstream FAK pathway, then elevated the transcriptional regulation of SOX6 on EMT and glycolysis-related genes’ promoter activity (Fig. 7c).

## Discussion

STC2 was first identified as a secreted glycosylated protein in endocrine gland of bony fish and involved in calcium and phosphate transportation (Ishibashi et al. 1998; Wagner et al. 1986). Recent studies have demonstrated that STC2 is associated with cell proliferation, EMT, and clinical progression in a variety of cancers. Although Horikawa et al. demonstrated that loss of STC2 enhanced cell death post-radiation and the expression of STC2 was upregulated by metabolic stress such as hypoxia in NPC (He et al. 2019), they did not explored the inner molecular mechanism. Moreover, they have only been validated in cell lines. However, the functions of STC2 and its underlying molecular mechanisms as a tumor activator in NPC remain unclear.

Actually, NPC stands out among head and neck tumors in aggressive and metastatic tendencies. Due to the importance of STC2 in cancer development and the imperfection of the previous studies, our experiment verified STC2 influence on the invasion ability and metabolism reprogramming in NPC and found a new pathway involved. In our study, we have revealed the function of STC2 in enhancing EMT and glycolysis traits in NPC and illustrated the underlying molecular mechanisms of such function STC2 knockdown inhibits cell expansion and tumorigenicity of NPC cells.

ITGB2 belongs to the integrin family, which encodes an integrin beta chain and combines with the alpha chains to form integrin heterodimers. Integrins are a kind of cell surface proteins involved in cell adhesion and cell surface–mediated signal transduction. FAK is one of the downstream of integrin beta, and has been reported in the acceptance of uterine adhesion to blastocysts. Activation of FAK is important for cell proliferation and intracellular signal transduction. Integrin-FAK signaling could activate a lot of signaling pathways through phosphorylation and protein interactions, thereby promoting tumorigenesis (Kumar et al. 2017; Leng et al. 2016; Li et al. 2018; Zhang et al. 2018). Liu et al. also showed that ITGB2 exerted the promotion effect on cancer metastasis and activated integrin-FAK pathway (Liu et al. 2018). In our study, STC2 was demonstrated to functionalize as the synergistic effector of ITGB2 to activate FAK pathway.

SOX6 is a member of Sex-determining region Y-box family which acts as a transcription factor involved in multiple stem/progenitor cells and embryonic development. Previous studies identified the importance of SOX in cancer development and metastasis (Kamachi and Kondoh 2013). For example, SOX6 suppressed tumor invasion by mediating the transcription of twist, a transcription factor involved in the promotion of EMT (Jiang et al. 2018). Our results showed that STC2 targets SOX6 by activating ITGB2/FAK, thereby regulating the transcription of the downstream of SOX6, which has significant impacts on cell proliferation, EMT, and glycolysis in NPC. To the best of our knowledge, our study is the first to reveal that STC2 silencing suppresses EMT and glycolysis by inhibiting ITGB2/FAK/SOX6 signaling in NPC.

In summary, we found that STC2 was highly expressed in primary nasopharyngeal carcinoma tissues and lymph node metastatic tissues. Silencing STC2 inhibited cell proliferative, invasion, and glycolysis. Moreover, RNA-seq analyses revealed that STC2 targets integrins and FAK signal. Furthermore, we demonstrated the direct interaction of ITGB2 with STC2. Collectively, our results demonstrated that STC2 promoted metastasis and glycolysis traits by regulating ITGB2/FAK/SOX6 axis, and suggest that the signaling axis might provide a novel therapeutic target for the inhibition of NPC progression and metastasis.

## Supplementary Information

ESM 1 (TIF 2434 kb)

High Resolution Image (PNG 7156 kb)

ESM 2 (TIF 347 kb)

High Resolution Image (PNG 2793 kb)

## Abbreviations

NPC: nasopharyngeal carcinoma
EBV: Epstein–Barr virus
EMT: epithelial-mesenchymal transition
STC2: stanniocalcin-2
ITGB2: integrin β2
FAK: focal adhesion kinase
SOX6: SRY-Box Transcription Factor 6
