# Supplementary figures and images for: Stanniocalcin-2 promotes cell EMT and glycolysis via activating ITGB2/FAK/SOX6 signaling pathway in nasopharyngeal carcinoma
Source: Cell Biol Toxicol. 2021 Apr 2;38(2):259–72. doi: 10.1007/s10565-021-09600-5 (PMC8986754; doi:10.1007/s10565-021-09600-5)

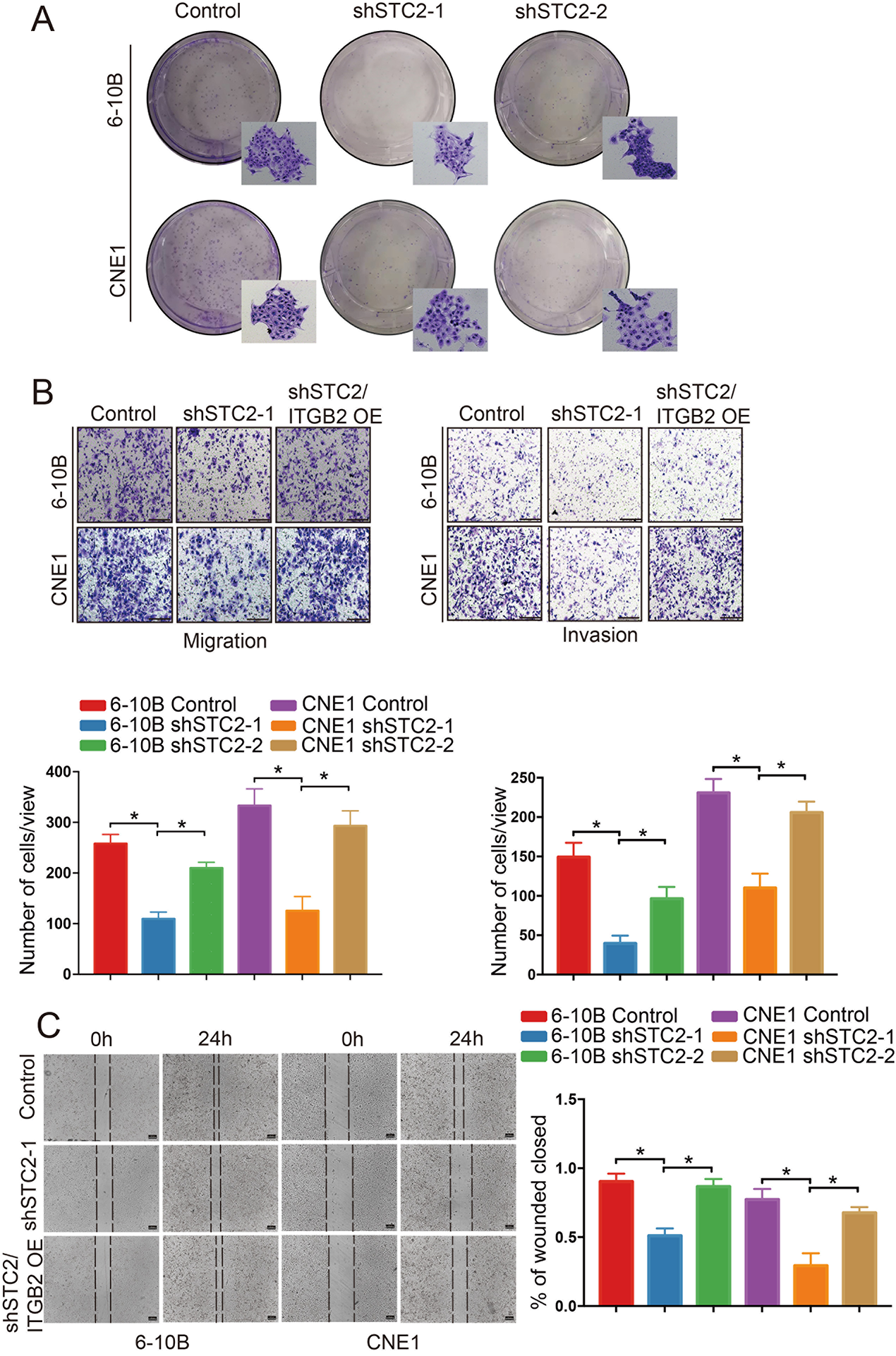

Supplement: Supplementary file 2 — High Resolution Image (PNG 7156 kb) [file 10565_2021_9600_Fig8_ESM.png]

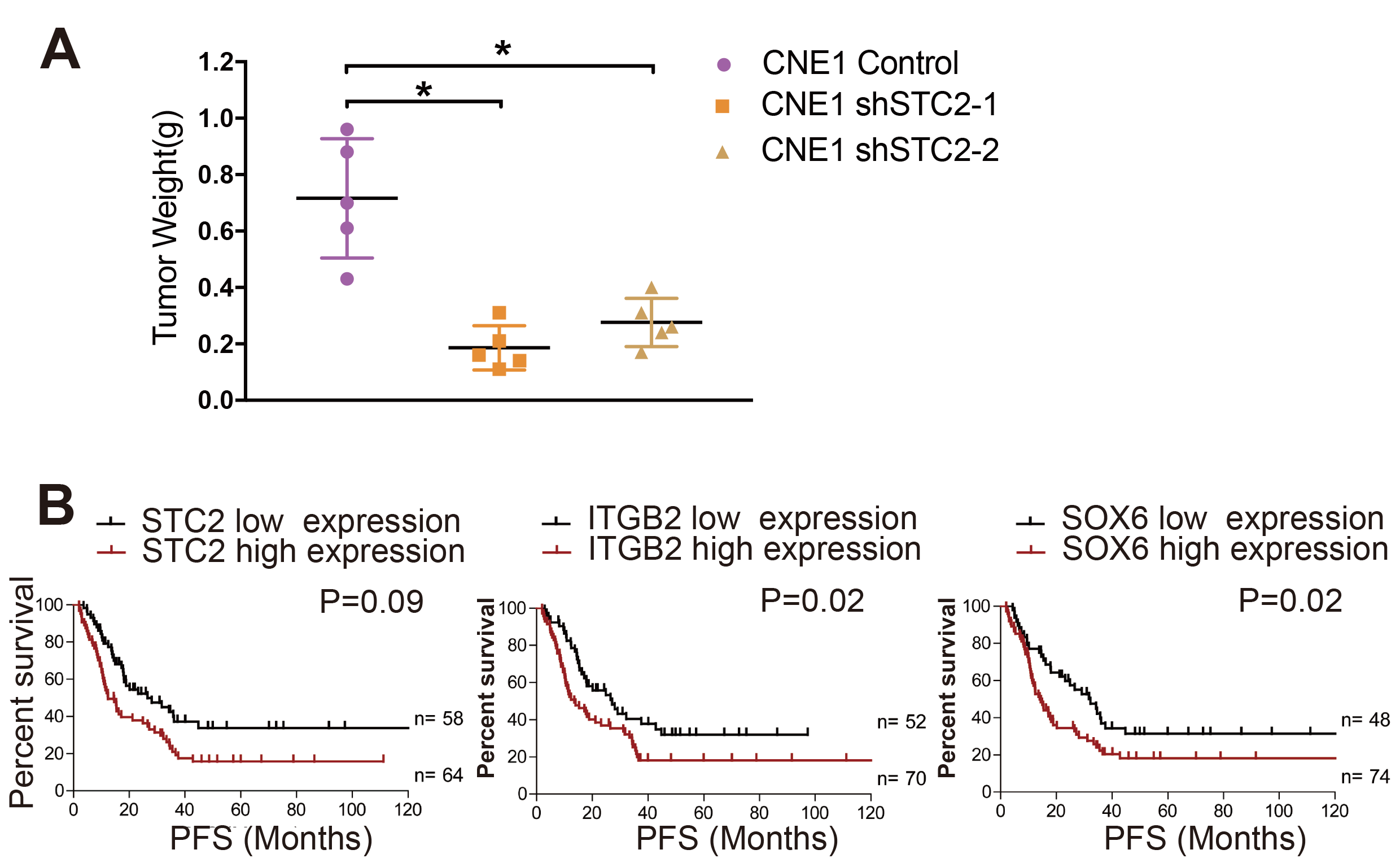

Supplement: Supplementary file 3 — (TIF 347 kb) [file 10565_2021_9600_MOESM2_ESM.tif]

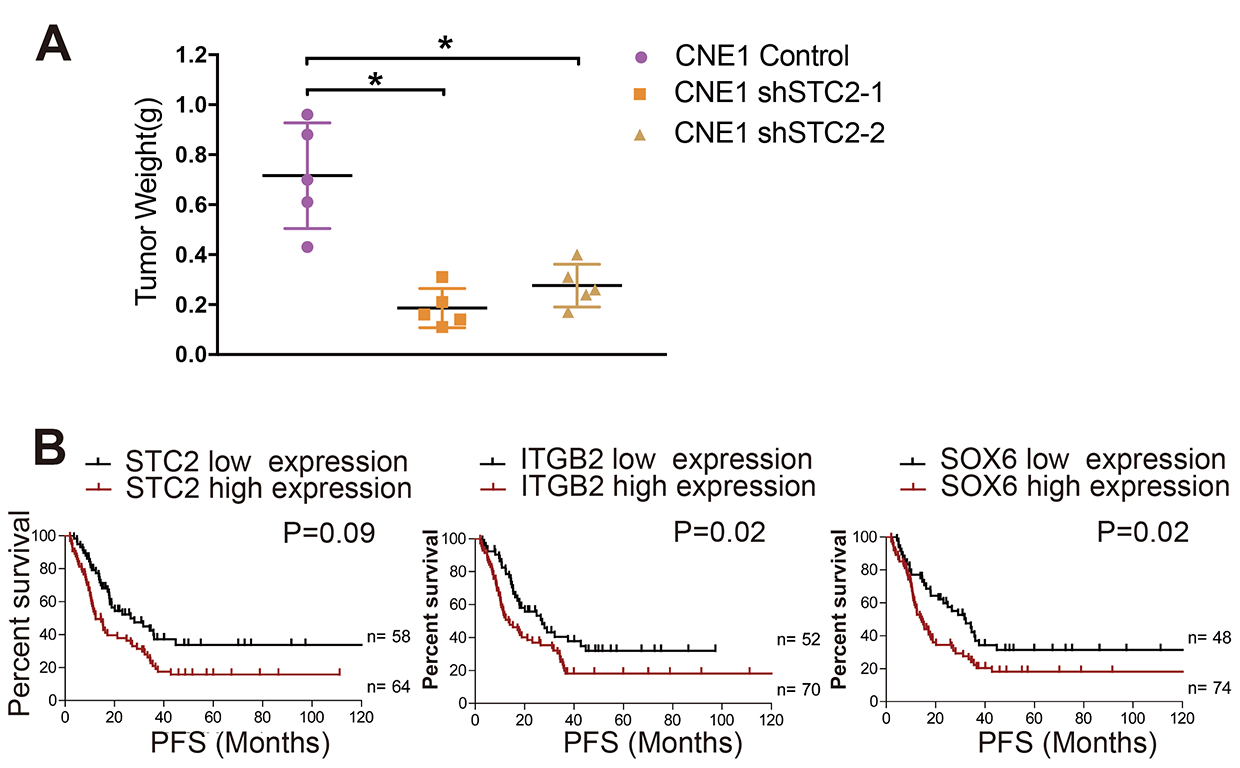

Supplement: Supplementary file 4 — High Resolution Image (PNG 2793 kb) [file 10565_2021_9600_Fig9_ESM.png]
